# Multiparametric MRI and auto-fixed volume of interest-based radiomics signature for clinically significant peripheral zone prostate cancer

**DOI:** 10.1007/s00330-019-06488-y

**Published:** 2019-11-27

**Authors:** Jeroen Bleker, Thomas C. Kwee, Rudi A. J. O. Dierckx, Igle Jan de Jong, Henkjan Huisman, Derya Yakar

**Affiliations:** 1 Medical Imaging Center, Departments of Radiology, Nuclear Medicine and Molecular Imaging, University Medical Center Groningen, University of Groningen, Hanzeplein 1, 9700 RB Groningen, The Netherlands; 2 Department of Urology, University Medical Center Groningen, University of Groningen, Hanzeplein 1, 9700 RB Groningen, The Netherlands; 3 Department of Radiology and Nuclear Medicine, Radboud University Medical Center, Geert Grooteplein Zuid 10, 6525 GA Nijmegen, The Netherlands

**Keywords:** Machine learning, Magnetic resonance imaging, Prostatic neoplasms, Neoplasm grading

## Abstract

**Objectives:**

To create a radiomics approach based on multiparametric magnetic resonance imaging (mpMRI) features extracted from an auto-fixed volume of interest (VOI) that quantifies the phenotype of clinically significant (CS) peripheral zone (PZ) prostate cancer (PCa).

**Methods:**

This study included 206 patients with 262 prospectively called mpMRI prostate imaging reporting and data system 3–5 PZ lesions. Gleason scores > 6 were defined as CS PCa. Features were extracted with an auto-fixed 12-mm spherical VOI placed around a pin point in each lesion. The value of dynamic contrast-enhanced imaging(DCE), multivariate feature selection and extreme gradient boosting (XGB) vs. univariate feature selection and random forest (RF), expert-based feature pre-selection, and the addition of image filters was investigated using the training (171 lesions) and test (91 lesions) datasets.

**Results:**

The best model with features from T2-weighted (T2-w) + diffusion-weighted imaging (DWI) + DCE had an area under the curve (AUC) of 0.870 (95% CI 0.980–0.754). Removal of DCE features decreased AUC to 0.816 (95% CI 0.920–0.710), although not significantly (*p* = 0.119). Multivariate and XGB outperformed univariate and RF (*p* = 0.028). Expert-based feature pre-selection and image filters had no significant contribution.

**Conclusions:**

The phenotype of CS PZ PCa lesions can be quantified using a radiomics approach based on features extracted from T2-w + DWI using an auto-fixed VOI. Although DCE features improve diagnostic performance, this is not statistically significant. Multivariate feature selection and XGB should be preferred over univariate feature selection and RF. The developed model may be a valuable addition to traditional visual assessment in diagnosing CS PZ PCa.

**Key Points:**

*• T2-weighted and diffusion-weighted imaging features are essential components of a radiomics model for clinically significant prostate cancer; addition of dynamic contrast-enhanced imaging does not significantly improve diagnostic performance.*

*• Multivariate feature selection and extreme gradient outperform univariate feature selection and random forest.*

*• The developed radiomics model that extracts multiparametric MRI features with an auto-fixed volume of interest may be a valuable addition to visual assessment in diagnosing clinically significant prostate cancer.*

**Electronic supplementary material:**

The online version of this article (10.1007/s00330-019-06488-y) contains supplementary material, which is available to authorized users.

## Introduction

Prostate cancer (PCa) is currently the most common cancer among men, and comprises approximately 20% of all cancers in the western world [1, 2]. Although most patients with PCa can be successfully treated [3], it is still responsible for an estimated 10% of all male cancer-related deaths in the western world. Early and accurate detection of clinically significant (CS) PCa is important to initiate treatment in a timely manner and improve patient outcome [3].

Current methods used for the detection of PCa vary per institution. Nevertheless, prostate-specific antigen (PSA) testing with digital rectal examination (DRE) followed by transrectal ultrasound (TRUS) biopsy is a widely used diagnostic algorithm. However, PSA testing suffers from a high number of false positives combined with a considerable number of false negatives [4]. The high false-positive rate leads to unnecessary TRUS biopsies. Furthermore, TRUS biopsies also suffer from sampling errors (i.e., both false negatives and underestimation of the true Gleason grade) [5]. The diagnostic limitations of PSA testing followed by TRUS biopsies lead to unnecessary patient discomfort, anxiety, and complications [6].

Multiparametric magnetic resonance imaging (mpMRI) has gained popularity as a non-invasive imaging technique for CS PCa detection and biopsy guidance that may overcome many of the shortcomings of the combination of PSA and TRUS alone [7–9]. Despite its potential, correct diagnosis of CS PCa based on mpMRI requires skill and experience. With the introduction of PI-RADS, and later PI-RADS v2, the diagnostic performance of radiologists has improved [8, 10]. Nevertheless, PI-RADS v2 is by no means a perfect system. Radiologists still need extensive experience to correctly discriminate CS from non-CS tumors [11, 12], with the additional issue that some lesions are not visible on mpMRI [13, 14]. Computer-aided diagnosis (CAD) aimed to increase correct diagnosis; however, due to the use of a small group of handcrafted features, its success is dependent on expert knowledge [15]. Therefore, there is a need for new technology that improves CS PCa detection on mpMRI without expert knowledge dependency. The use of radiomics, which aims to extract relevant quantitative tumor features from imaging data that may be unperceivable by the human eye, may fill this void [16].

A limited number of studies already aimed to find such quantitative mpMRI radiomics features for CS PCa [17–19]. However, these previous studies suffered from several methodological shortcomings, including small sample sizes (as low as 30 patients), heterogeneous datasets mixing peripheral zone (PZ) with transition zone (TZ) tumors, manual delineation of tumor suspicious regions (which introduces observer dependency and decreases model generalization), and a very small number of initial quantitative features that were explored (as low as 10 features). Furthermore, no previous radiomics study investigated whether the use of dynamic contrast-enhanced (DCE, k-trans) sequences adds useful diagnostic information to a radiomics-based approach. Finally, no research has been performed on whether the multivariate-based diagnosis of CS PCa on mpMRI works better with multivariate feature selection and extreme gradient boosting (XGB) [20, 21] than the recommended univariate selection and random forest (RF) [22].

The aim of this study was to create a model based on mpMRI radiomics features extracted from an auto-fixed volume of interest (VOI) that quantifies the phenotype of CS PZ PCa.

## Materials and methods

### Patient data

This study was institutional review board approved, and all patients provided informed consent for the original dataset creation. The data used for this study was originally part of the ProstateX dataset [23]. A total of 206 patients from this dataset were scanned at the Radboud University Medical Center (Nijmegen, the Netherlands) in 2012, and these patients comprised the present study population. Patients in the ProstateX dataset had a median PSA level of 13 ng/ml (range 1 to 56 ng/ml) with a median age of 66 (range 48 to 83 years) [24]. The mpMRI protocol was performed on a 3.0-T MRI scanner (MAGNETOM Trio or Skyra, Siemens Healthcare); see Table 1 for a summary of applied sequences (more detailed information can be found in the previously published challenge) [25]. All patients in this study underwent mpMRI of the prostate because of at least one previous negative systematic TRUS prostate biopsy and persistent clinical suspicion of PZ PCa (i.e., elevated PSA and/or abnormal DRE). These patients had a total of 262 prospectively called PI-RADS 3–5 PZ lesions that were subsequently subjected to in-bore MRI targeted biopsy, which was used as reference standard (all under the supervision of a highly experienced radiologist in prostate mpMRI, > 20 years of experience). PZ lesions with a Gleason score of > 6 (International Society of Urological Pathology (ISUP) grade group ≥ 2) were defined and labeled as CS PCa, while PZ lesions with a Gleason score of ≤ 6, with normal or benign histopathology results (e.g., prostatitis, benign prostatic hyperplasia, or prostatic intraepithelial neoplasia), were labeled as the non-CS category.

**Table 1:** Summary of sequences used for mpMRI of the prostate

Sequence	T2-weighted imaging Turbo spin echo	Dynamic contrast-enhanced imaging 3D turbo gradient echo	Diffusion-weighted single-shot echo-planar imaging
In-plane resolution (mm)	0.5	1.5	2
Slice thickness (mm)	3.6	4	3.6
Temporal resolution (s)		3.5	
Sequence orientation	Axial, sagittal, and coronal	Axial	Axial
Additional remarks	No endorectal coil	No endorectal coil Used for K-trans calculation	No endorectal coil *b*-values of 50, 400, and 800 s/mm^2^ Used for calculated *b*-value of 1400 s/mm^2^ and mono-exponentially calculated apparent diffusion coefficient map

*mpMRI* multiparametric MRI

### Training and test dataset

Radiomics features [16] for the training dataset were calculated from prostate mpMRI scans of 130 patients who had a total of 171 prospectively called PI-RADS 3–5 PZ lesions, of which 35 proved to be CS PZ PCa and 136 were grouped in the non-CS PZ category according to MRI targeted biopsy results. Importantly, the test data set (which consisted of 76 patients with 91 prospectively called PI-RADS 3–5 PZ lesions, of which 20 were CS PCa and 71 were non-CS PZ entities) was kept separate from the training set and remained untouched until the development of the model, to avoid a biased result [26].

### Auto-fixed segmentation

An auto-fixed tumor VOI was used for the extraction of the radiomics features in order to increase their reproducibility and robustness [27]. By using identical VOIs placed in the same manner, observer variability and dependency can be reduced. Originally, the prospectively called PI-RADS 3–5 PZ lesions were marked with a pin point in the visually most aggressive part of the lesion (area with the lowest apparent diffusion coefficient (ADC) value). Marking of this visually most aggressive part of the PZ lesion was performed under the supervision of an expert prostate radiologist (> 20 years of experience). Future clinical implementation of a model with auto-fixed segmentation requires the user to manually perform the marking. Scanner coordinates corresponding with the supervised marking were stored and converted to image coordinates. In this study, we then automatically created a spherical VOI with the lesion image coordinates at its center. The raster geometry package (Python Software Foundation) was used for the spherical volume calculation. For each of the image directions, a radius was calculated based on VOI size and image voxel spacing. The auto-fixed VOI size was set to 12 mm in order to sufficiently cover most prostate lesions which have an average diameter of 10 mm [28]. For a number of patients in the ProstateX dataset, deviations were discovered from the dimensional information reported in Table 1. Interpolation of these voxels was omitted due to uncertainty about the interpolation size and technique for mpMRI [29]. Solving these uncertainties for each mpMRI sequence requires a large number of experiments which is outside the scope of the current article. Additionally, no issues were expected due to the equal representation of the deviations in both the training and test datasets and the fact that feature calculation was based on a collection of voxels.

### Radiomics features extraction

**Fig. 1:**
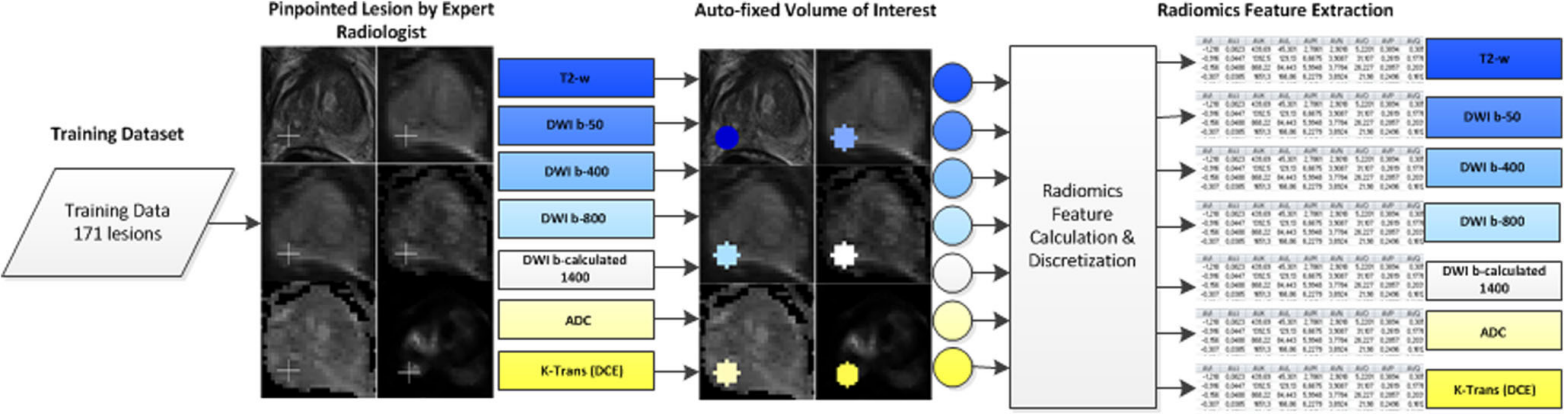
Schematic pipeline for the extraction of radiomics features from mpMRI data. ADC = apparent diffusion coefficient map, DCE = dynamic contrast-enhanced, DWI = diffusion-weighted imaging, T2-w = T2 weighted

Ninety-two quantitative radiomics features which comprised six different feature types were calculated in Python using Pyradiomics [30]. Eighteen first-order features which use basic statistics to characterize the voxel intensity distribution, 23 gray level co-occurrence matrix features (GLCM), 16 gray level run length matrix features (GLRLM), 16 gray level size zone matrix features (GLSZM), 14 gray level dependence matrix features (GLDM), and 5 neighboring gray tone difference matrix features (NGTDM) were used to quantify the image texture in the VOI. Previous work by Aerts et al and Zwanenburg et al provide full feature names and their mathematical descriptions [16, 29]. Pixels used for the calculation of the 80 texture features were discretized in fixed gray level bins (for further details, see supplemental digital content 1). An overview of the radiomics feature extraction pipeline is given in Fig. 1.

### Extreme gradient boosting, expert-based feature pre-selection, and the use of filters

Due to the uncertain complimentary role of DCE imaging for the diagnosis of CS PCa [31, 32], an additional analysis was performed to determine the effect of DCE features in the radiomics approach. A total of two different mpMRI training datasets were created (Table 2). The first mpMRI dataset consisted of T2-weighted (T2-w) imaging, diffusion-weighted imaging (DWI) with *b*-values of 50, 400, 800, calculated 1400 s/mm^2^, and an calculated ADC map (abbreviated as T2-w + DWI). The second mpMRI dataset expanded on this with DCE imaging, k-trans (abbreviated as T2-w + DWI + DCE). For each of the two mpMRI training datasets, two radiomics models were created. One of these models used a previously suggested machine learning approach for radiomics [22] with a combination of univariate feature selection and RF classifiers. In an effort to improve this, we first introduced another model based on a combination of multivariate feature selection and XGB classifiers. This can be considered a good fit for high-dimensional tabular data like in radiomics [20, 21]. Both univariate and multivariate feature selection aim to find features with strong relationships with the output labels (CS PCa, non-CS entities). Multivariate feature selection also takes relationships between features into account. Detailed information about the machine learning approach can be found in supplemental digital content 2. Second, we investigated whether expert-based feature pre-selection could increase the performance of the radiomics model [27]. Feature selection was performed by a specialized uro-radiologist (D.Y.) with 5 years of experience in mpMRI of the prostate. The selection was based on clinical experience and domain knowledge [33]; selected quantitative features were thought to correspond to clinical characteristics of CS PZ PCa or the non-CS category. Third, we investigated whether the use of image filters (e.g., edge enhancement and voxel intensity enhancement) improved the diagnostic accuracy of our model. Previous research has shown that applying certain image filters before feature extraction can enhance certain lesion type differences and improve diagnosis [34–38]. Detailed filter descriptions and their effect can be found in supplemental digital content 3. Using the best combination of mpMRI dataset (T2-w + DWI vs. T2-w + DWI + DCE), machine learning approach (RF vs. XGB), with or without expert-based feature pre-selection, and the effect of features taken from filtered images (e.g., edge enhancement), different models were created.

**Table 2:** Summary of mpMRI dataset composition

mpMRI dataset 1	mpMRI dataset 2
T2-weighted imaging (axial, sagittal, and coronal planes)	T2-weighted imaging (axial, sagittal, and coronal planes)
Diffusion-weighted imaging (*b*-values of 50, 400, 800, and calculated *b*-value of 1400 s/mm^2^)	Diffusion-weighted imaging (*b*-values of 50, 400, 800, and calculated *b*-value of 1400 s/mm^2^)
Calculated ADC map	Calculated ADC-map
	K-trans (axial plane, calculated from DCE imaging)

*ADC* apparent diffusion coefficient map, *DCE* dynamic contrast-enhanced, *mpMRI* multiparametric MRI

### Statistical analysis

Each developed model was used to create an area under the curve (AUC) score based on 10 × 10-fold receiver operating curves (ROCs) on the training data. Training AUCs were checked for normality using Shapiro-Wilk’s test and compared using the Wilcoxon signed rank test [39, 40]. Additionally, all models from the different experiments were evaluated on the separate test dataset. ROCs were created with corresponding AUCs and 95% confidence intervals (CI) created with 5000 times bootstrapping. AUCs were compared using 5000 times bootstrapping. Statistical analyses were performed using R version 3.5.2 software (R Foundation for Statistical Computing) with the pROC package [41].

## Results

### Effect of DCE on radiomics

**Fig. 2:**
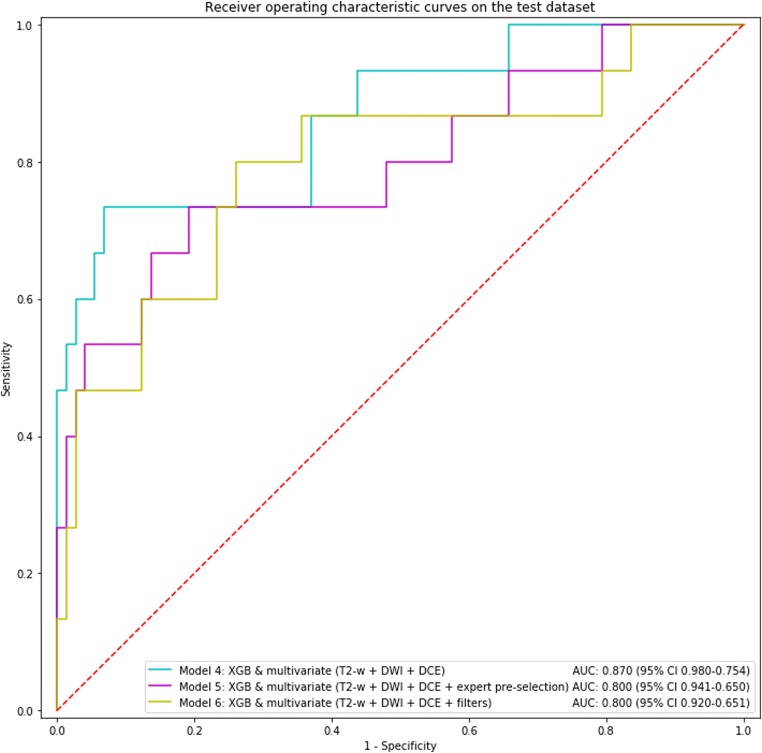
Test dataset receiver operating curves (ROCs) for models 1 to 4 based on mpMRI dataset 1 (T2-w + DWI) and mpMRI dataset 2 (T2-w + DWI + DCE). Model 1 (blue, mpMRI dataset 1) and model 2 (green, mpMRI dataset 2) curves are created by a combination of univariate feature selection and a random forest (RF) classifier. The curves for model 3 (red, mpMRI dataset 1) and model 4 (cyan, mpMRI dataset 2) were created using multivariate feature selection and extreme gradient boosting (XGB)

**Fig. 3:**
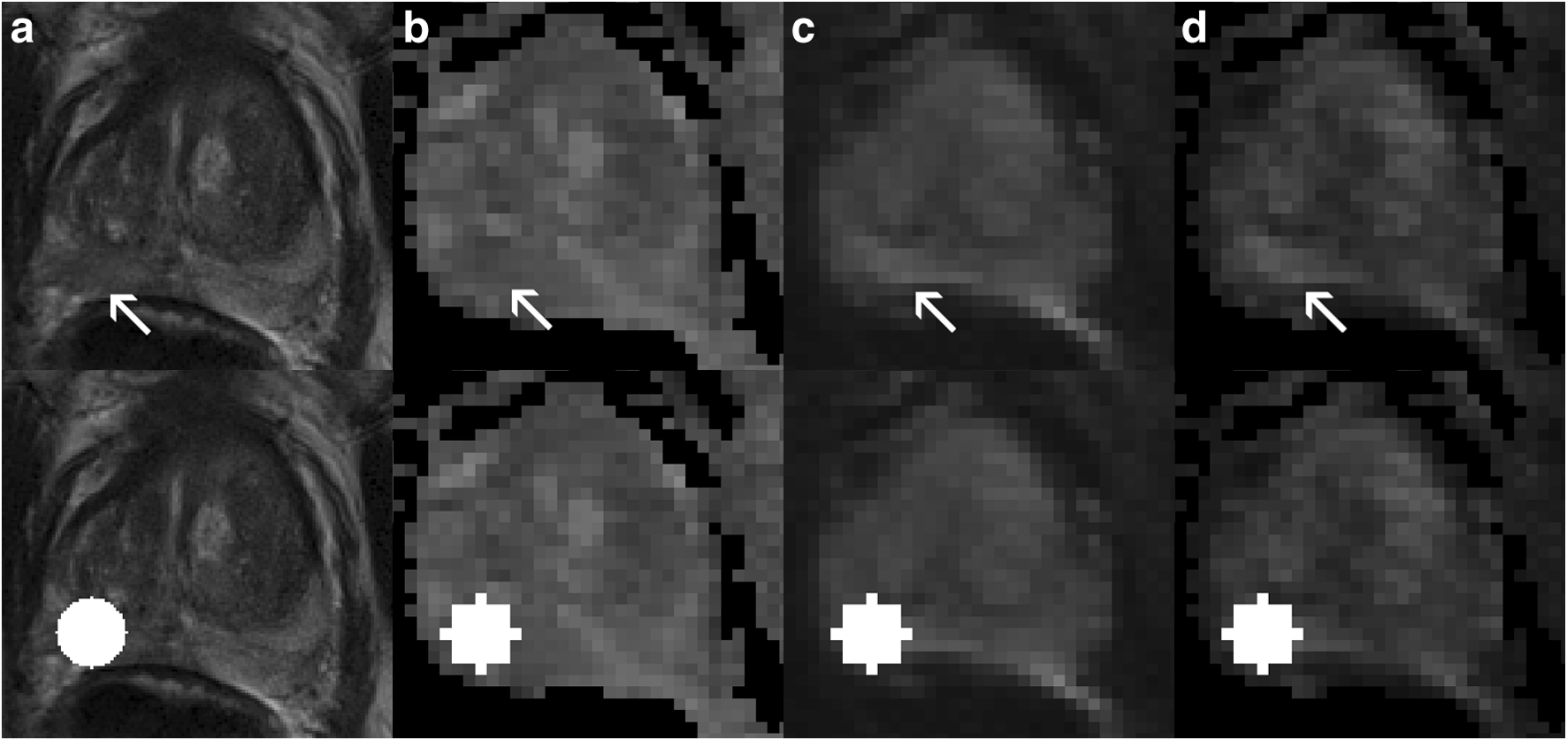
True-positive example for model 3 (T2-w + DWI) which predicted a clinically significant (CS) prostate cancer (PCa) lesion. This patient had a peripheral zone (PZ) lesion (classifiable as PI-RADS 4) which was pinpointed (the visually most aggressive part) originally by an expert (arrow, first row), which proved to be CS PCa (Gleason score > 6). **a** T2-w (axial), **b** ADC, **c** DWI *b*-value 800 s/mm^2^, **d** DWI calculated *b*-value 1400 s/mm^2^. Second row, segmentations using the auto-fixed volume of interest (VOI, marked in white) were placed around the visually most aggressive lesion pinpoint

**Fig. 4:**
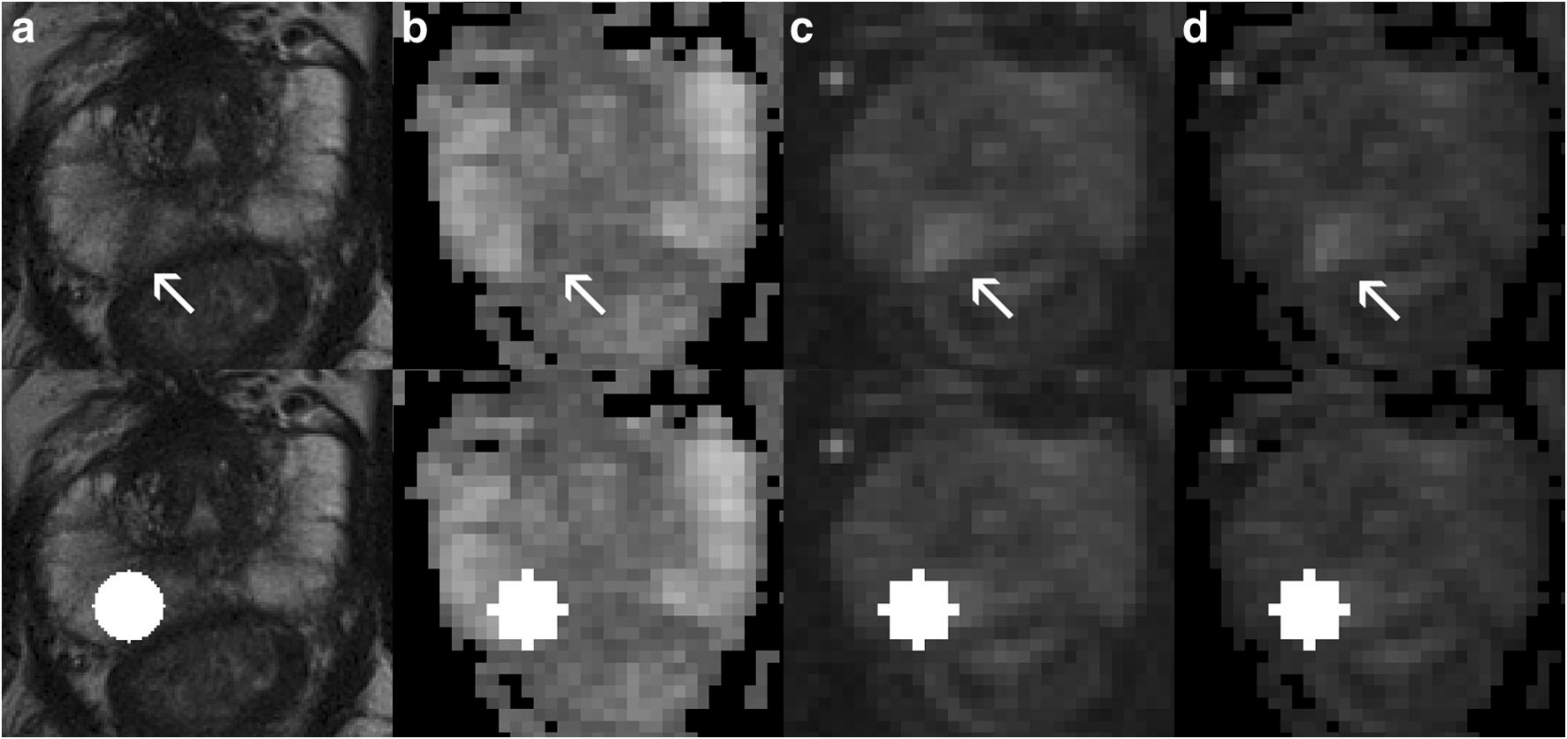
False-positive example for model 3 (T2-w + DWI) which predicted a clinically significant (CS) prostate cancer (PCa) lesion. This patient had a peripheral zone (PZ) lesion (classifiable as PI-RADS 4) which was pinpointed (the visually most aggressive part) originally by an expert (arrow, first row), which proved to be a non-CS entity (Gleason score < 6). **a** T2-w (axial), **b** ADC, **c** DWI *b*-value 800 s/mm^2^, **d** DWI calculated *b*-value 1400 s/mm^2^. Second row, segmentations using the auto-fixed volume of interest (VOI, marked in white) were placed around the visually most aggressive lesion pinpoint

The comparison of models based on the two different mpMRI datasets (T2-w + DWI vs. T2-w + DWI + DCE) showed that the addition of DCE imaging did lead to a significant improvement on the training dataset (*p* < 0.001, Table 3). This significant improvement found in the training dataset did not translate to the test dataset for both RF and XGB (AUC 0.780 vs. 0.745 *p* = 0.657, AUC 0.870 vs. 0.816 *p* = 0.119). ROCs for the test dataset of the models are given in Fig. 2, with corresponding AUCs in Table 5. The best scoring model from Table 5, AUC 0.870 (95% CI 0.980–0.754), sensitivity 0.86 (63/73), and specificity 0.73 (11/15), takes a shared first place when compared to the original 71 entries and the over 200 ongoing entries in the ProstateX challenge [23, 42], which was the original purpose of the data used in this study. Figure 3 gives an evaluation example for model 3 (XGB + T2-w + DWI, AUC: 0.816, sensitivity 0.75 (55/73), and specificity 0.67 (10/15)) which was predicted correctly while Fig. 4 shows an example of a false positive.

**Table 3:** AUCs for training mpMRI dataset 1 and mpMRI dataset 2 using the different machine learning approaches univariate and RF vs. multivariate and XGB, including the different mpMRI sequences from where the features selected by univariate or multivariate selection originate

	Approach	Initial mpMRI dataset	Feature selection mpMRI sequence origin	AUCs	Comparisons*
Model 1	RF and univariate	(T2-w + DWI)	DWI calculated *b*-value of 1400 s/mm^2^	0.762 (95% CI 0.790–0.740)	M2–M1: *p* < 0.001
Model 2	RF and univariate	(T2-w + DWI + DCE)	DCE k-trans	0.850 (95% CI 0.870–0.824)	M2–M1: *p* < 0.001 M4–M2: *p* = 0.003
Model 3	XGB and multivariate	(T2-w + DWI)	T2-w, DWI (*b*-value of 800 and calculated *b*-value of 1400 s/mm^2^), ADC	0.850 (95% CI 0.874–0.830)	M4–M3: *p* < 0.001
Model 4	XGB and multivariate	(T2-w + DWI + DCE)	T2-w, DWI (*b*-value of 800 and calculated *b*-value of 1400 s/mm^2^), ADC, DCE	0.890 (95% CI 0.903–0.870)	M4–M3: *p* < 0.001 M4–M2: *p* = 0.003

*AUCs* area under the curves, *CI* confidence interval, *DWI* diffusion-weighted imaging, *DCE* dynamic contrast-enhanced, *M* model, *mpMRI* multiparametric MRI, *RF* random forest, *T2-w* T2-weighted, *XGB* extreme gradient boosting

*Comparisons were made using the Wilcoxon signed rank test

### Multivariate selection and XGB versus univariate selection and RF

**Fig. 5:**
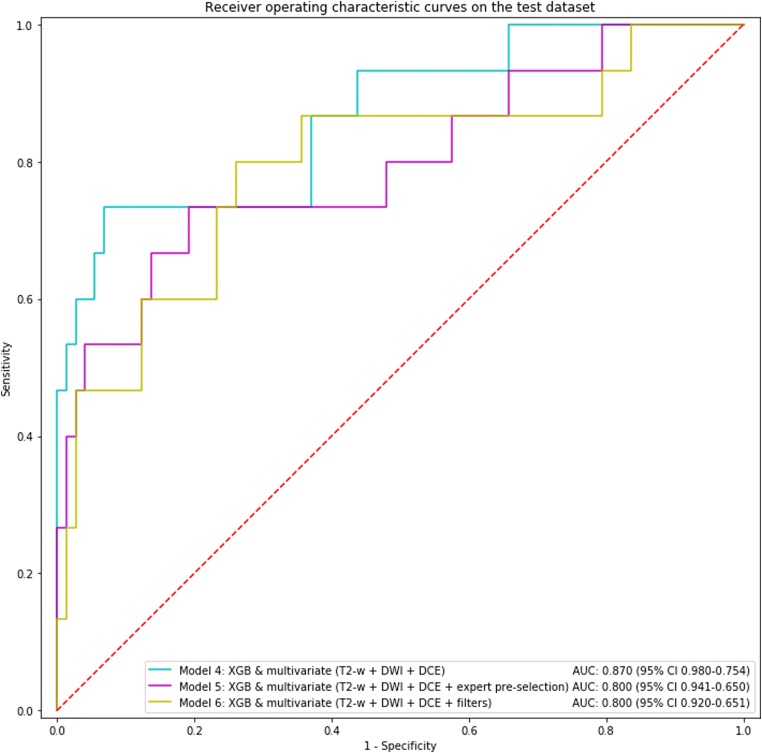
Test dataset ROCs for model 4 (cyan, mpMRI dataset 2, T2-w + DWI + DCE and repeated from Fig. 2), model 5 (magenta, T2-w + DWI + DCE + expert pre-selection), and model 6 (yellow, T2-w + DWI + DCE + filters (supplemental digital content 3)) based on multivariate selection and XGB

For both the mpMRI datasets defined in Table 2, the combination of multivariate feature selection and an XGB classifier achieved significantly higher AUCs when compared to univariate selection and RF (*p* = 0.003, Table 3). Of note, the features selected by univariate selection (strongest relation with the labels, CS PCa vs. non-CS entities) originate from a single mpMRI sequence, while multivariate selection features are selected from multiple sequences. When applied to the test dataset, the models based on multivariate feature selection and XGB outperformed the models based on univariate selection and RF (AUC 0.870 vs. 0.780 *p* = 0.028). ROCs for these models are given in Fig. 5, with corresponding AUCs in Table 5.

### Expert-based pre-selection and filtered images

The XGB model based on the best performing mpMRI dataset (T2-w + DWI + DCE), performed significantly better than the model which used expert-based feature pre-selection (XGB + T2-w + DWI + DCE + expert-based pre-selection; *p* < 0.001, Table 4). On the test dataset, there was no significant difference between both models (AUC 0.870 vs. 0.800 *p* = 0.273, Fig. 4 and Table 5). Adding features taken from filtered images (supplemental digital content 3) to this best performing dataset (XGB + T2-w + DWI+ DCE+ filters) did not lead to an improvement when compared to the XGB model (XGB + T2-w + DWI+ DCE, *p* = 0.208). The results on the test dataset did not show a significant improvement either (AUC 0.870 vs. 0.800, *p* = 0.177, Fig. 4 and Table 5).

**Table 4:** AUCs and the origin of features selected by multivariate selection for the addition of expert pre-selection and image filters by using the best performing mpMRI training dataset with the best machine learning approach (T2-w + DWI + DCE, multivariate and XGB)

	Approach	Initial mpMRI dataset	Feature selection mpMRI sequence origin	AUCs	Comparisons*
Model 5	XGB and multivariate	(T2-w + DWI + DCE) + expert pre-selection	T2-w, DWI calculated *b*-value of 1400 s/mm^2^, ADC, DCE	0.800 (95% CI 0.823–0.775)	M4–M5: *p* < 0.001
Model 6	XGB and multivariate	(T2-w + DWI + DCE) + filters	T2-w, DWI (*b*-value of 800 and calculated *b*-value of 1400 s/mm^2^), ADC, DCE	0.871 (95% CI 0.894–0.850)	M4–M6: *p* = 0.208

*AUCs* area under the curves, *CI* confidence interval, *DWI* diffusion-weighted imaging, *M* model, *mpMRI* multiparametric MRI, *RF* random forest, *T2-w* T2- weighted, *XGB* extreme gradient boosting

*Comparisons were made using the Wilcoxon signed rank test

**Table 5:** AUCs with bootstrapping for models 1 to 6 on the separate test dataset

	Approach	mpMRI dataset	AUCs	Comparisons*
Model 1	RF and univariate	(T2-w + DWI)	0.745 (95% CI 0.890–0.602)	M2–M1: *p* = 0.657
Model 2	RF and univariate	(T2-w + DWI + DCE)	0.780 (95% CI 0.900–0.661)	M2–M1: *p* = 0.657 M4–M2: *p* = 0.028
Model 3	XGB and multivariate	(T2-w + DWI)	0.816 (95% CI 0.920–0.710)	M4–M3: *p* = 0.119
Model 4	XGB and multivariate	(T2-w + DWI + DCE)	0.870 (95% CI 0.980–0.754)	M4–M3: *p* = 0.119 M4–M2: *p* = 0.028
Model 5	XGB and multivariate	(T2-w + DWI + DCE) + expert pre-selection	0.800 (95% CI 0.941–0.650)	M4–M5: *p* = 0.273
Model 6	XGB and multivariate	(T2-w + DWI + DCE) + filters	0.800 (95% CI 0.920–0.651)	M4–M6: *p* = 0.177

*AUCs* area under the curves, *CI* confidence interval, *DWI* diffusion-weighted imaging, *M* model, *mpMRI* multiparametric MRI, *RF* random forest, *T2-w* T2-weighted, *XGB* extreme gradient boosting

*Comparisons were made using 5000 times bootstrapping

## Discussion

Our best scoring model uses a combination of mpMRI features taken from T2-w, DWI, and DCE imaging, extracted with an auto-fixed VOI, and achieved a relatively high AUC of 0.870 (95% CI 0.980–0.754) in the test dataset. Nevertheless, we found that the addition of features from DCE did not lead to a significantly improved radiomics model compared to features taken from T2-w and DWI alone. Furthermore, a combination of multivariate feature selection and XGB was found to be the best machine learning approach, while expert-based feature pre-selection and the addition of features taken from filtered images did not lead to a significant improvement. Importantly, we used datasets with prospectively called PI-RADS 3–5 lesions, in which the overall detection rate of CS PCa is known to be only 55% [12]. Therefore, our results indicate that the developed model may provide additional diagnostic value and might potentially reduce the number of unnecessary biopsies.

Interestingly, we found that the addition of features taken from DCE imaging did not lead to a significant increase in diagnostic test performance (*p* = 0.119). This is in line with and supports the current trend of omitting DCE imaging from the routine MRI protocol and using the so-called biparametric MRI (bpMRI) to decrease study time and costs [43]. For routine prostate examinations, there is no difference in diagnostic performance between mpMRI and bpMRI [31, 32]. However, our results show a non-significant increase in diagnostic performance for models that did include DCE features. This non-significant increase might be explained using PI-RADSv2.1 which identifies five special patient scenarios where mpMRI should be preferred over bpMRI [44]. Our results also show that multivariate feature selection and XGB should be preferred over univariate feature selection and an RF classifier (AUC of the latter, 0.780 (95% CI 0.900–0.661), *p* = 0.028). This contradicts the results of a previous study by Parmar et al [22] that reported univariate feature selection and an RF classifier to be the best machine learning approach for radiomics [22]. This contradiction may be due to the different data types used, since Parmar et al [22] used computed tomography instead of mpMRI. Furthermore, our results showed that the univariate feature selection tends to focus on a single sequence, suggesting a good correlation between the single sequence of concern and the differentiation between CS PZ PCa and non-CS entities. However, given the fact that multivariate selection performed significantly better and did not focus on a single sequence, it appears that feature redundancies between features taken from a single sequence that are not tested in univariate selection diminish the performance of the model. Including expert-based pre-selection of radiomics features did not lead to a significant change in performance (AUC 0.800 (95% CI 0.941–0.650), *p* = 0.273). Though interestingly, it did lead to the least difference between the training and test datasets. A possible explanation for this finding may be that pre-selection based on clinical experience and domain knowledge eliminated the least reproducible features [27]. However, due to the loss in performance on the training dataset, the approach in which a single radiologist selects features based on experience and knowledge might not be viable and more research should be performed. The inclusion of features extracted from filtered mpMRI images, which should theoretically enhance lesion differences, did not significantly improve results (AUC 0.800 (95% CI 0.920–0.651), *p* = 0.177). This finding is in contrast to previous studies [35, 37, 45] and may be explained by the use of a broad selection of multiple filter types while relying on the feature selection algorithms rather than domain knowledge. However, further investigation is needed before fully dismissing them.

There are a number of other studies that aimed to build an mpMRI radiomics model that quantifies the phenotype of CS PZ PCa [18, 19, 46]. Although it is difficult to fully compare the quantitative features we found with earlier research, e.g., due to different patient populations and variations in imaging protocols, some comparison between the present results and previous studies can be made. A recent study by Bonekamp et al [19] compared a radiomics model with the mean ADC and radiologist assessment for the diagnosis of CS PCa lesions. However, the approach used for the development of their radiomics model was limited by manual tumor lesion delineation and mixing of both PZ and TZ lesions. Not unimportantly, quantitative ADC measurements have a limited role in clinical practice. This is due to the variety of acquisition and analysis methodologies that do not allow for comparison of ADC values between centers and establishment of universally useful diagnostic cut-off values [47, 48]. Furthermore, manually delineating tumor boundaries is prone to making results observer dependent (besides being labor intensive) and mixing PZ and TZ lesions ignores the fact that both types of PCa are phenotypically different [19, 49–51]. Another study by Khalvati et al [18] proposed a radiomics model which used a set of radiomics features with some statistical and textural features that partly matched our selection. Nevertheless, they did not investigate all mpMRI sequences such as DCE imaging and validated their radiomics model on a very small dataset of only 30 patients again without separating PZ from TZ lesions. Finally, a study by Xu et al introduced a radiomics model based on bpMRI radiomics features and a small set of clinical parameters [46]. Besides identical limitations to the ones mentioned above (manual delineation, mixing PZ and TZ lesions), Xu et al created a test dataset based on the date of the study instead of a random division. This, in combination with the observation that their test scores were higher than the then training scores, raises bias concerns. Additionally, they did not include a high calculated *b*-value which we found to be essential for models 1, 3, and 4 (Table 3).

The present study had several limitations. First, its results are only applicable to PZ lesions, and the model does not hold up for lesions in the TZ. TZ lesions, which are phenotypically different [49–51], should be investigated separately with the use of a dedicated model. Second, our study focused on lesion characterization and not on automatic detection of lesions suspicious of PCa. A recently published study [52] investigated an automatic detection system for PCa lesions prior to a radiologist’s interpretation. The authors of that study concluded that such a system introduced more false positives than a radiologist [52]. This raises the question of whether such automatic detection systems are suited for clinical practice at the moment. Third, due to the retrospective nature of the present study, mpMRI protocols were heterogeneous and performed on two different MRI systems. On the other hand, these differences yielded more diverse data that may actually have helped to increase reproducibility of the radiomics features [27]. Nevertheless, to be able to say with certainty that the model, and by extension the set of quantifying radiomics features, exhibit proper generalization, external validation should be performed in future studies. Finally, all patients underwent in-bore MRI targeted biopsy, whereas prostatectomy may have served as a better reference standard. However, this reflects clinical practice, and only including patients who had undergone prostatectomy could have introduced selection bias [53].

In conclusion, the phenotype of CS PZ PCa lesions can be quantified using a radiomics approach based on features extracted from T2-w + DWI using an auto-fixed VOI. Although DCE features improve diagnostic performance, this is not statistically significant. Multivariate feature selection and XGB should be preferred over univariate feature selection and RF. The developed model may be a valuable addition to traditional visual assessment in diagnosing CS PZ PCa.

## Electronic supplementary material

ESM 1 (DOCX 37 kb)

## Abbreviations

2D LBP: Two-dimensional local binary pattern
ADC: Apparent diffusion coefficient
AUC: Area under the curve
CI: Confidence interval
CS: Clinically significant
DCE: Dynamic contrast-enhanced
DRE: Digital rectal examination
DWI: Diffusion-weighted imaging
GLCM: Gray level co-occurrence matrix
GLDM: Gray level dependence matrix
GLRLM: Gray level run length matrix
GLSZM: Gray level size zone matrix
H: High-pass filter
ISUP: International Society of Urological Pathology
L: Low-pass filter
LoG: Laplacian of Gaussian
mpMRI: Multiparametric magnetic resonance imaging
NGTDM: Neighboring gray tone difference matrix features
PCa: Prostate cancer
PI-RADS: Prostate imaging reporting and data system
PZ: Peripheral zone
RF: Random forest
ROC: Receiver operating curve
T2-w: T2-weighted imaging
TZ: Transition zone
VOI: Volume of interest
XGB: Extreme gradient boosting
