# Supplementary material for: Multiparametric MRI and auto-fixed volume of interest-based radiomics signature for clinically significant peripheral zone prostate cancer
Source: Eur Radiol. 2019 Nov 27;30(3):1313–24. doi: 10.1007/s00330-019-06488-y (PMC7033141; doi:10.1007/s00330-019-06488-y)
Supplement: Supplementary file 1 — (DOCX 37 kb) [file 330_2019_6488_MOESM1_ESM.docx]

**Supplemental Digital Content**

**Supplemental Digital Content 1. Radiomics features fixed bin size discretization**

To improve reproducibility of promising quantitative features, the voxel intensities of the VOIs were discretized for the calculation of all texture features.^1^ Fixed bin size discretization was used since it has been proven to be relevant for reproducibility and feature comparison.^2^ When using fixed bin discretization, a single bin represents a gray level range set by the bin width. Bin width was chosen in such a way that the total number of bins ended up somewhere between 30 and 130.^3^ All images were first normalized and later discretized using a bin width of 0.15, with the exception of 3D LBP that used 0.05.

**Supplemental Digital Content 2. Machine learning approaches**

The machine learning approach recommended for radiomics by Parmar et al. ^4^ was reproduced starting with Wilcoxon based univariate feature selection. The Wilcoxon scoring function was taken from the appendix of the original article.^4^ The optimal number of selected features which were fed to the random forest model was tuned using randomized search 5 fold cross validation.^5^ Likewise, the hyperparameters for the random forest classifier were tuned.

Another machine learning approach was taken based on multivariate feature selection in an effort to improve on the aforementioned combination of univariate selection and random forest. Multivariate feature selection takes relations between features into account while univariate selection only focuses on the relation between a single feature and the prediction labels.^6^ Joint mutual information maximization (JMIM) was selected as multivariate technique because it was found to mostly outperform other often used multivariate techniques during its introduction.^4,5,7,8^ One such technique it outperformed was minimum redundancy maximum relevance (mRMR), a multivariate technique with proven effectiveness in radiomics.^4^ JMIM uses mutual information and tries to find features that have a high relevance for distinguishing CS PCa from non-CS entities while reducing redundant features. While the maximum of the minimum criterion ensures it does not suffer from incorrect removal of redundant features and overestimation of the relevance of which mRMR and other multivariate techniques suffer.^7^

The random forest classifier was replaced by extreme gradient boosting (XGB), a tree boosting system often preferred by Kaggle machine learning competition winners for structured datasets.^9^ Extreme gradient boosting is based on regular boosting and tries to find a number of weak decision trees and combine these into an ensemble. Finding the optimal multivariate feature selection technique, number of features selected by this technique and overall hyperparameter setting for XGB was done using randomized search in 5 fold cross validation.

**Supplemental Digital Content 3. Image filters and enhancement**

Laplacian of Gaussian (LoG) filters were applied to the original mpMR images of both training datasets (D1: T2-weighted imaging, DWI b-values 50, 400, 800, 1400 mm/s^2^, ADC; D2: T2-weighted imaging, DWI b-values 50, 400, 800, 1400 mm/s^2^, ADC, and DCE) to emphasize the detection of areas inside the VOI where voxels differ and rapidly change in intensity. Noise was first suppressed by a Gaussian filter which was followed by a Laplacian for edge detection.^10^ Different Gaussian filter width distances between 0.5 mm and 2 mm were used to find the most effective distance. Smaller distances focus on voxels over a shorter distance, while higher distances put emphasis on intensity changes over longer distances. Additionally, a gradient filter was used to find the gradient magnitude for each individual voxel. This gave information about the change in image intensity gradient in a certain direction, which adds useful information for the detection and diagnosis of certain lesions.^11^ A multilevel discrete wavelet decomposition was performed in two dimensions (x,y) to emphasize naturally occurring directional features on the mpMR images. Four decompositions were yielded using Discrete Meyer wavelets.^12^ The decompositions consisted of all combinations of the wavelet used as low (L) or high (H) pass filter (LL, LH, HL, HH) in each of the directions. The low pass filter gives an approximation of the mpMR image structures while the high pass filter focuses on image details. Furthermore, a uniform two-dimensional local binary pattern (2D LBP) was calculated for the voxels inside the VOI. The rotationally invariant uniform local binary patterns have shown to be a valuable addition to different classification problems like facial recognition. ^13–15^ Lastly, one image enhancement filters that takes the logarithm of each pixel intensity was applied. An additional factor which uses the maximum image intensity is used by the enhancement filters to enhance potential differences between CS lesions and non-CS entities.

**Supplemental Digital Content 4. Top 20 most influential features for model 1 to 6 supplied by the Random forest and XGB algorithms**

Scores supplied by either the random forest algorithm or the extreme gradient boosting algorithm. Summation of all importance scores lead to 1, higher scores mean higher influence on the end result.

| **Importance** | **Top 20 Features Model 1** | **Importance** | **Top 20 Features Model 2** |
| --- | --- | --- | --- |
| 0,142694 | original_glcm_MCC-DWI-bcal | 0,074413 | original_glcm_DifferenceAverage-K-Trans |
| 0,098962 | original_ngtdm_Strength-DWI-bcal | 0,065561 | original_glcm_DifferenceEntropy-K-Trans |
| 0,079893 | original_glcm_Imc1-DWI-bcal | 0,063078 | original_ngtdm_Coarseness-K-Trans |
| 0,063151 | original_firstorder_RobustMeanAbsoluteDeviation-DWI-bcal | 0,060663 | original_glcm_Id-K-Trans |
| 0,059041 | original_glszm_GrayLevelVariance-DWI-bcal | 0,051059 | original_glcm_Idm-K-Trans |
| 0,053515 | original_glcm_ClusterTendency-DWI-bcal | 0,050967 | original_firstorder_Range-K-Trans |
| 0,051436 | original_glrlm_GrayLevelVariance-DWI-bcal | 0,046135 | original_firstorder_Maximum-K-Trans |
| 0,050534 | original_gldm_GrayLevelVariance-DWI-bcal | 0,035738 | original_firstorder_90Percentile-K-Trans |
| 0,037092 | original_firstorder_Range-DWI-bcal | 0,034792 | original_firstorder_RootMeanSquared-K-Trans |
| 0,035206 | original_firstorder_Variance-DWI-bcal | 0,033971 | original_gldm_DependenceNonUniformity-K-Trans |
| 0,034331 | original_ngtdm_Complexity-DWI-bcal | 0,031041 | original_firstorder_MeanAbsoluteDeviation-K-Trans |
| 0,034259 | original_glcm_SumSquares-DWI-bcal | 0,028738 | original_gldm_DependenceNonUniformityNormalized-K-Trans |
| 0,031103 | original_firstorder_Maximum-DWI-bcal | 0,027709 | original_glcm_SumAverage-K-Trans |
| 0,030456 | original_glcm_DifferenceVariance-DWI-bcal | 0,027098 | original_glcm_JointAverage-K-Trans |
| 0,030256 | original_glrlm_RunEntropy-DWI-bcal | 0,024484 | original_firstorder_RobustMeanAbsoluteDeviation-K-Trans |
| 0,029919 | original_firstorder_90Percentile-DWI-bcal | 0,024056 | original_glcm_SumEntropy-K-Trans |
| 0,028511 | original_firstorder_MeanAbsoluteDeviation-DWI-bcal | 0,023488 | original_gldm_SmallDependenceEmphasis-K-Trans |
| 0,026092 | original_glszm_SizeZoneNonUniformity-DWI-bcal | 0,023361 | original_firstorder_Entropy-K-Trans |
| 0,025101 | original_firstorder_InterquartileRange-DWI-bcal | 0,022458 | original_glszm_ZoneEntropy-K-Trans |
| 0,022072 | original_glcm_InverseVariance-DWI-bcal | 0,022273 | original_glszm_ZonePercentage-K-Trans |

| **Importance** | **Top 20 Features Model 3** | **Importance** | **Top 20 Features Model 4** |
| --- | --- | --- | --- |
| 0,051059 | original_glcm_Correlation-T2-sag | 0,063448 | original_gldm_LargeDependenceHighGrayLevelEmphasis-T2-sag |
| 0,041096 | original_gldm_LargeDependenceHighGrayLevelEmphasis-T2-sag | 0,06069 | original_glcm_Correlation-T2-sag |
| 0,038605 | original_glcm_Idmn-ADC | 0,048276 | original_ngtdm_Coarseness-K-Trans |
| 0,036115 | original_glszm_GrayLevelNonUniformity-T2-tra | 0,048276 | original_firstorder_Range-T2-tra |
| 0,032379 | original_firstorder_Range-T2-tra | 0,038621 | original_gldm_LargeDependenceHighGrayLevelEmphasis-ADC |
| 0,028643 | original_firstorder_Median-ADC | 0,037241 | original_glcm_Idn-ADC |
| 0,028643 | original_ngtdm_Complexity-T2-tra | 0,037241 | original_glszm_GrayLevelNonUniformity-DWI-bcal |
| 0,027397 | original_ngtdm_Strength-DWI-bcal | 0,031724 | original_glcm_MCC-DWI-bcal |
| 0,026152 | original_ngtdm_Busyness-DWI-b800 | 0,031724 | original_firstorder_Mean-ADC |
| 0,026152 | original_glszm_SmallAreaEmphasis-DWI-bcal | 0,028966 | original_glcm_MCC-DWI-b800 |
| 0,024907 | original_firstorder_Minimum-T2-cor | 0,028966 | original_firstorder_RobustMeanAbsoluteDeviation-DWI-bcal |
| 0,022416 | original_glszm_GrayLevelNonUniformityNormalized-DWI-bcal | 0,027586 | original_ngtdm_Busyness-K-Trans |
| 0,021171 | original_glcm_MCC-DWI-b800 | 0,027586 | original_glcm_Imc2-ADC |
| 0,019925 | original_glcm_Correlation-DWI-b800 | 0,026207 | original_firstorder_Minimum-T2-cor |
| 0,01868 | original_glcm_MCC-T2-sag | 0,024828 | original_firstorder_10Percentile-ADC |
| 0,017435 | original_firstorder_RobustMeanAbsoluteDeviation-DWI-bcal | 0,023448 | original_glszm_SizeZoneNonUniformityNormalized-K-Trans |
| 0,017435 | original_firstorder_Mean-ADC | 0,022069 | original_ngtdm_Strength-DWI-bcal |
| 0,017435 | original_glcm_MCC-DWI-bcal | 0,022069 | original_glcm_Imc1-DWI-b800 |
| 0,017435 | original_firstorder_Energy-T2-cor | 0,02069 | original_glszm_ZoneEntropy-DWI-bcal |
| 0,016189 | original_firstorder_Kurtosis-ADC | 0,017931 | original_firstorder_TotalEnergy-K-Trans |

| **Importance** | **Top 20 Features Model 5** | **Importance** | **Top 20 Features Model 6** |
| --- | --- | --- | --- |
| 0,044888 | original_glcm_Idmn-ADC | 0,053704 | logarithm_firstorder_Range-T2-tra |
| 0,0399 | original_gldm_LargeDependenceHighGrayLevelEmphasis-T2-sag | 0,048148 | log-sigma-1-5-mm-3D_glrlm_LongRunHighGrayLevelEmphasis-T2-sag |
| 0,038653 | original_firstorder_Median-ADC | 0,044444 | original_firstorder_Median-ADC |
| 0,03616 | original_ngtdm_Strength-DWI-bcal | 0,040741 | log-sigma-0-5-mm-3D_glcm_Correlation-DWI-bcal |
| 0,033666 | original_glszm_LowGrayLevelZoneEmphasis-ADC | 0,040741 | log-sigma-0-5-mm-3D_firstorder_Mean-DWI-bcal |
| 0,033666 | original_firstorder_Mean-T2-cor | 0,038889 | log-sigma-1-5-mm-3D_firstorder_90Percentile-ADC |
| 0,032419 | original_firstorder_Minimum-ADC | 0,038889 | logarithm_firstorder_Maximum-DWI-bcal |
| 0,028678 | original_firstorder_Minimum-T2-cor | 0,037037 | logarithm_glszm_GrayLevelNonUniformity-DWI-b800 |
| 0,028678 | original_firstorder_Kurtosis-ADC | 0,031481 | original_glszm_ZoneEntropy-DWI-bcal |
| 0,026185 | original_glszm_GrayLevelNonUniformity-T2-tra | 0,031481 | logarithm_gldm_DependenceEntropy-DWI-bcal |
| 0,023691 | original_firstorder_Kurtosis-T2-sag | 0,02963 | logarithm_glcm_MCC-DWI-bcal |
| 0,021197 | original_glszm_GrayLevelNonUniformityNormalized-DWI-bcal | 0,027778 | original_gldm_DependenceEntropy-DWI-bcal |
| 0,021197 | original_ngtdm_Strength-T2-tra | 0,025926 | original_firstorder_Minimum-ADC |
| 0,021197 | original_firstorder_Range-T2-tra | 0,024074 | original_glszm_GrayLevelNonUniformity-DWI-b800 |
| 0,021197 | original_glszm_GrayLevelNonUniformity-DWI-b800 | 0,024074 | logarithm_glszm_SmallAreaEmphasis-DWI-bcal |
| 0,01995 | original_glcm_Contrast-DWI-bcal | 0,024074 | log-sigma-1-5-mm-3D_ngtdm_Busyness-DWI-bcal |
| 0,01995 | original_firstorder_Energy-ADC | 0,022222 | original_firstorder_10Percentile-ADC |
| 0,018703 | original_firstorder_Uniformity-DWI-b800 | 0,022222 | logarithm_glszm_GrayLevelNonUniformity-DWI-bcal |
| 0,017456 | original_glszm_GrayLevelNonUniformity-DWI-bcal | 0,02037 | log-sigma-1-5-mm-3D_glcm_ClusterTendency-DWI-b800 |
| 0,017456 | original_firstorder_Kurtosis-DWI-bcal | 0,02037 | original_glszm_GrayLevelNonUniformity-DWI-bcal |

1. Zwanenburg A, Leger S, Vallières M, et al. Image biomarker standardisation initiative. 2016;(November). Available at: http://arxiv.org/abs/1612.07003.

2. Leijenaar RTH, Nalbantov G, Carvalho S, et al. The effect of SUV discretization in quantitative FDG-PET Radiomics: The need for standardized methodology in tumor texture analysis. *Sci. Rep.* 2015;5(August):1–10.

3. Van Griethuysen JJM, Fedorov A, Parmar C, et al. Computational radiomics system to decode the radiographic phenotype. *Cancer Res.* 2017;77(21):e104–e107.

4. Parmar C, Grossmann P, Bussink J, et al. Machine Learning methods for Quantitative Radiomic Biomarkers. *Sci. Rep.* 2015;5:1–11.

5. Ding C, Peng H. Minimum Redundancy Feature Selection from Microarray Gene Expression Data. *Comput. Syst. Bioinformatics. CSB2003. Proc. 2003 IEEE Bioinforma. Conf. CSB2003*. 2005;3(2):523–528.

6. Saeys Y, Inza I, Larrañaga P. A review of feature selection techniques in bioinformatics. *Bioinformatics*. 2007;23(19):2507–2517.

7. Bennasar M, Hicks Y, Setchi R. Feature selection using Joint Mutual Information Maximisation. *Expert Syst. Appl.* 2015;42(22):8520–8532.

8. Peng H, Long F, Ding C. Feature selection based on mutual information: Criteria of Max-Dependency, Max-Relevance, and Min-Redundancy. *IEEE Trans. Pattern Anal. Mach. Intell.* 2005;27(8):1226–1238.

9. Chen T, Guestrin C. XGBoost: A Scalable Tree Boosting System. In: *Knowledge Delivery and Data mining*.; 2016. Available at: http://arxiv.org/abs/1603.02754%0Ahttp://dx.doi.org/10.1145/2939672.2939785.

10. Chen JS, Huertas A, Medioni G. Fast convolution with Laplacian-of-Gaussian masks. *IEEE Trans. Pattern Anal. Mach. Intell.* 1987;PAMI-9:584–590.

11. Thawani R, McLane M, Beig N, et al. Radiomics and radiogenomics in lung cancer: A review for the clinician. *Lung Cancer*. 2018;115(October 2017):34–41.

12. Bartušek K, Přinosil J, Smékal Z. Wavelet-based de-noising techniques in MRI. *Comput. Methods Programs Biomed.* 2011;104(3):480–488.

13. Ojala T, Pietikäinen M, Harwood D. A comparative study of texture measures with classification based on featured distributions. *Pattern Recognit.* 1996;29(1):51–59.

14. Ahonen T, Hadid A, Pietikäinen M. Face recognition with local binary patterns. *Comput. vision-eccv 2004*. 2004:469–481.

15. Barkan O, Weill J, Wolf L, et al. Fast high dimensional vector multiplication face recognition. *Proc. IEEE Int. Conf. Comput. Vis.* 2013:1960–1967.
